# The Role of Imaging in Radiation Therapy Planning: Past, Present, and Future

**DOI:** 10.1155/2014/231090

**Published:** 2014-04-10

**Authors:** Gisele C. Pereira, Melanie Traughber, Raymond F. Muzic

**Affiliations:** ^1^Department of Radiation Oncology, University Hospitals Case Medical Center, Case Western Reserve University, Cleveland, OH 44106, USA; ^2^Philips Healthcare, MR Therapy, Cleveland, OH, USA; ^3^Case Center for Imaging Research, Case Western Reserve University, Cleveland, OH, USA; ^4^Department of Radiology, University Hospitals Case Medical Center, Case Western Reserve University, Cleveland, OH 44106, USA

## Abstract

The use of ionizing radiation for cancer treatment has undergone extraordinary development during the past hundred years. The advancement of medical imaging has been critical in helping to achieve this change. The invention of computed tomography (CT) was pivotal in the development of treatment planning. Despite some disadvantages, CT remains the only three-dimensional imaging modality used for dose calculation. Newer image modalities, such as magnetic resonance (MR) imaging and positron emission tomography (PET), are also used secondarily in the treatment-planning process. MR, with its better tissue contrast and resolution than those of CT, improves tumor definition compared with CT planning alone. PET also provides metabolic information to supplement the CT and MR anatomical information. With emerging molecular imaging techniques, the ability to visualize and characterize tumors with regard to their metabolic profile, active pathways, and genetic markers, both across different tumors and within individual, heterogeneous tumors, will inform clinicians regarding the treatment options most likely to benefit a patient and to detect at the earliest time possible if and where a chosen therapy is working. In the post-human-genome era, multimodality scanners such as PET/CT and PET/MR will provide optimal tumor targeting information.

## 1. Introduction to Radiation Therapy

The three most important aspects of cancer treatment are surgery, chemotherapy (in earlier times referred to simply as medicine), and radiation therapy. Of these, surgery is the oldest with records discovered by Edwin Smith, an American Egyptologist, and describing the surgical treatment of cancer in Egypt circa 1600 B.C. [1]. Medicines were also used in ancient Egypt at the time of the pharaohs, although the use of chemotherapy in cancer was first used in the early 1900s by the German chemist, Paul Ehrlich [2]. In contrast, radiation therapy, the therapeutic use of ionizing radiation, is by far the most recent technique used to treat cancer. X-rays, a kind of ionizing radiation, were discovered in 1895 by Wilhelm Roentgen and within months were used to treat tumors. This use of ionizing radiation has undergone extraordinary development during the past century. As we will discuss, the advancements in medical imaging have been critical to the evolution of modern radiation therapy.

In the late nineteenth century, three discoveries regarding ionizing radiation were instrumental in the development of radiation therapy:

1. November 8, 1895: X-rays discovered by Wilhelm Conrad Roentgen (1845–1923);
2. March 1, 1896: radioactivity discovered by Henri Becquerel (1852–1908);
3. December 26, 1898: radium discovered by Madame Curie (Maria Sktodowska) (1867–1934).

There are two, general classes of radiation therapy: brachytherapy and teletherapy. “Brachy,” a Greek word, means short distance and “tele” means long distance. Brachytherapy is treatment performed by placing the radioactive source near or in contact with a tumor, that is, the use of intracavitary or intraluminal placement of the treatment source. Conversely, teletherapy is treatment with the radioactive source at a distance from the patient/tumor. Teletherapy, also known as external beam radiation therapy, may be classified by the voltage applied to produce X-ray photons as in Table 1 [3].

The lower energy beams, produced by X-ray tubes, are well suited for diagnostic imaging. However, the use of these lower energy beams is limited in radiation therapy. Because these beams are highly attenuated, that is, poorly penetrating, the treatment of deep tumors results in an excessive radiation dose to the skin, thus limiting the ability to deliver curative doses, and so are of limited clinical usefulness. In order to treat deep tumors while maintaining a lower radiation skin dose, high-energy beams with greater penetrating power are required.

High-energy gamma-ray photons are emitted during radioactive decay of certain radionuclides. (X-rays and gamma-rays are both photons and produce the same interactions with tissue. They differ according to their origin. Gamma-rays originate from the changes between energy levels in the nucleus, while X-rays originate from changes between energy levels in the electrons orbiting the nucleus.) External-beam radiation therapy units may be created by collimating the gamma-ray emissions from large quantities of radioactive material. Examples of these units are teleradium utilizing Radium-226 and emitting gamma-rays with average energy of 1.2 MeV, telecesium utilizing Cesium-137 and emitting gamma-rays with average energy of 0.66 MeV, and the Cobalt-60 unit emitting gamma-rays at 1.17 MeV and 1.33 MeV. All of these units require storage of large amounts of radioactive materials and have associated radiation safety concerns, including securing the materials so that they are not used for terrorist activities. Therefore, the use of radioactive materials has largely been replaced by megavoltage machines.

The first megavoltage machine used to produce therapeutic X-rays was a type of accelerator called the Van de Graaff generator. It is an electrostatic accelerator which accelerates charged particles, in this case electrons. The high-energy electrons strike a target to create X-rays from 1 to 2 MeV in energy. Its first clinical use was at Huntington Memorial Hospital in Boston when it was installed in 1937.

Another type of accelerator used in radiation therapy is a betatron which has a hollow, doughnut shape with an alternating magnetic field to accelerate electrons. These accelerators were used specifically for the production of therapeutic electron beams, rather than X-rays. Most of these units were installed in European medical centers, had electron energies up to 50 MeV, and were used to treat deeply located tumors.

Van de Graaff generators and betatrons were the precursors of linear accelerators or “linacs.” Linacs to be used for radiation therapy were developed during and after World War II and used the high-frequency and high-power microwave sources developed for the manufacture of radar systems. Linacs accelerate charged particles, most commonly electrons, to create therapeutic electron or X-ray beams up to 25 MeV in energy. Electrons achieve acceleration by traveling through a high electronic field in a magnetic field that causes the electrons to take a spiral path of increasing radius. X-rays are produced when these accelerated electrons collide with a high-atomic-number target. In 1953, the first isocentric linac was installed at the Christie Hospital in Manchester, United Kingdom, and these units continue to be the mainstay of modern radiation therapy. The chronology of the development of linear accelerators for radiotherapy use is as follows:

1953: first isocentric linac installed, United Kingdom;
1954: first dual-throttle (X-rays and electrons) linac installed, St Bartholomew's Hospital, London;
1973: first dual-photon-energy linac installed, Antoni van Leeuwenkoek, Amsterdam;
1981: introduction of motorized collimators;
1985: new series of fully computer-controlled linacs established, thus allowing the development of modern radiotherapy of high complexity.

## 2. Past: Introduction of Imaging in Radiation Therapy Planning

**Figure 1:**
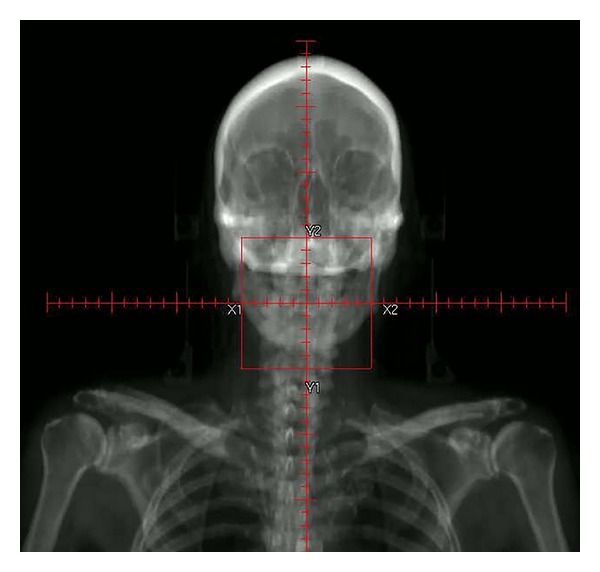
Creation of digital reconstructed radiographs or DRRs from CT.

Together with the progress of radiation therapy linacs was the development of dose calculation and treatment-planning techniques. The ability to quantify the delivered dose evolved from simple skin erythema observations in the late 1800s to single-point calculations inside the patient in the mid 1900's and to computer-based dose calculations in the late 1960s. Finally, with the invention of computed tomography (CT) by Cormack and Hounsfield in 1972, three-dimensional (3D) dose calculation became possible. The use of CT in treatment planning allowed several important advances in radiation therapy and resulted in greater precision in dose distribution, dose optimization, and patient positioning. However, one of the most important advances CT provided for 3D dose calculation was the precise visualization of the geometric positions of tumor and normal tissue in a patient. The radiation dose could then be calculated and optimized in order to determine the best dose distribution in the target (tumor), thus avoiding the surrounding normal tissue. Another advance CT offered was the creation of digitally reconstructed radiographs or DRRs (Figure 1) for patient position verification at the time of treatment using the linac.

## 3. Present: What Is Used in Practice Today

Currently, radiation therapy linacs are fully controlled by computers and with new techniques of dose delivery, such as intensity-modulated radiation therapy (IMRT), volumetric-modulated arc therapy (VMAT), and imaged-guided radiotherapy (IGRT), the treatment delivery precision is measured in millimeters [4]. A more precise method of target definition is necessary for such precise delivery. While CT has revolutionized the field of radiation therapy, further improvements in imaging are desirable in which the dose can be delivered with yet increased accuracy. CT has several limitations such as suboptimal tissue contrast, lack of functional information, and the inability to visualize small groups of cancer cells that are separated from the gross tumor. If we can overcome these limitations, we can further improve the precision of the target definition and provide better patient outcomes [5].

**Figure 2:**
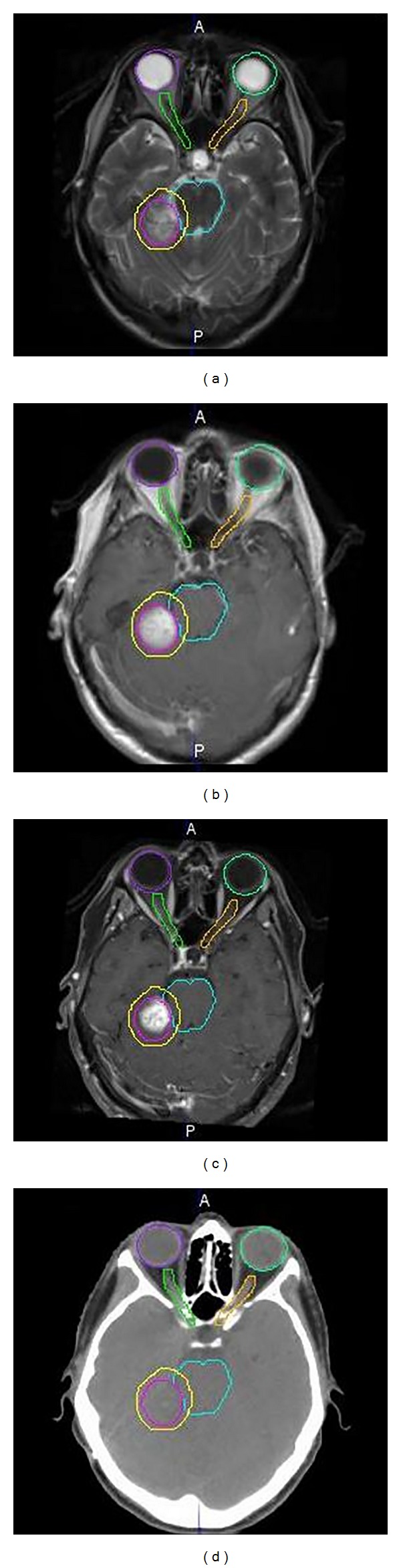
Differences in the soft-tissue contrast from four images. (a) is a T2-weighted MR image, (b) is a T1-weighted MR image, (c) is a T1-weighted MR image with contrast, and (d) is a CT image. The ROIs shown above are eyes (purple and light green colors), optical nerves (green and yellow colors), brain stem (teal color), gross tumor (pink color), and clinical target (yellow color) which extend from the gross tumor by a few millimeters with the intent of treating the subclinical microscopic extension of disease.

**Figure 3:**
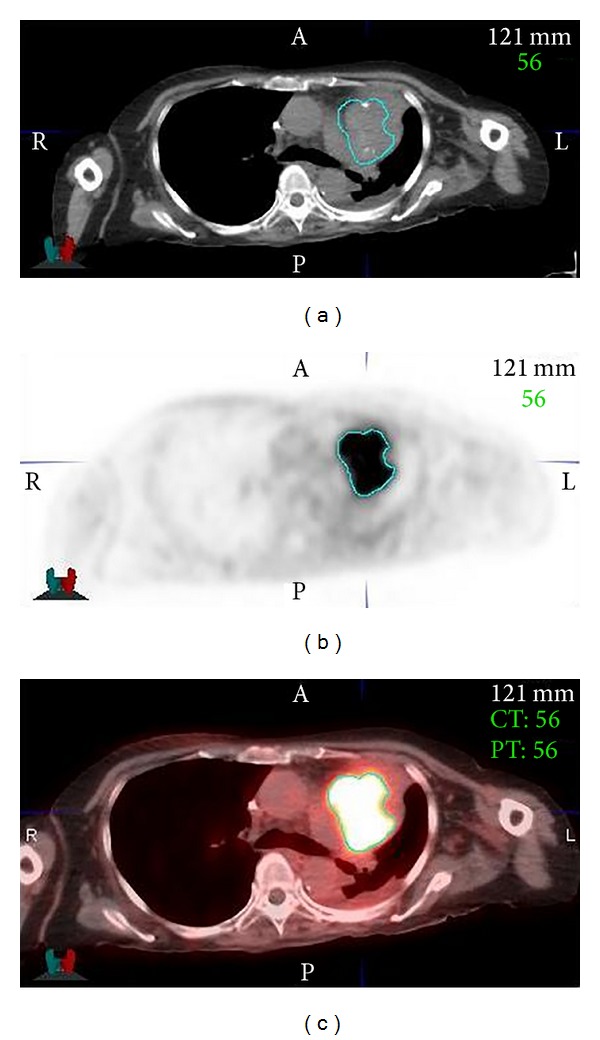
PET provides the ability to differentiate areas of neoplastic, hyper-metabolic activity within surrounding normal tissue where (a) is the CT image, (b) is the PET, and (c) is the fused PET/CT. All of the images show the gross tumor delineated from the PET image in teal color.

The application of other imaging modalities, such as magnetic resonance (MR) imaging and positron emission tomography (PET), can provide additional information in order to more precisely define tumor localization for treatment planning using radiation therapy [6–8]. In particular, MR has better soft-tissue contrast than CT and provides better visual discrimination between tissue that should be treated and that which should not (Figure 2) [9, 10]. PET allows the identification of areas of metabolic activity and thus allows the radiation oncologist to escalate the radiation dose for the most aggressively growing tumors or regions therein [11, 12] (Figure 3). Despite its limitations, for several reasons CT is currently the only 3D imaging method accepted for treatment planning. Most treatment-planning algorithms were developed specifically for CT as it was the first available 3D imaging modality and CT scanners are more commonly used than MR or PET. Furthermore, the geometric fidelity of CT is better than that of MR in which distortions may occur, and as CT generally has shorter acquisition times than MR or PET, organ/tumor motion management can be assessed. Most importantly, with CT it is possible to identify the mass attenuation coefficient (*μ*/*ρ* (m^2^/Kg)) or attenuation characteristics for high-energy photons, X-rays, and gamma-rays, as this is critical for precise dose calculation.

**Figure 4:**
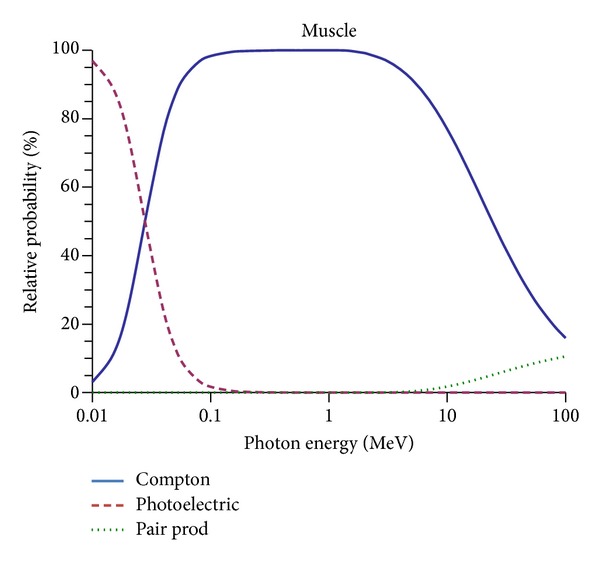
Probability of photon interaction (Compton, Photoelectric, and Pair Production) in muscle.

Photon interactions with tissue, such as photo-electric absorption, Compton scatter, and pair production, are dependent on the atomic number, electron density of the tissue, and photon energy (Figure 4). Therefore, to accurately calculate the radiation dose, the specific mass attenuation factor for different types of tissue, that is, heterogeneity, encountered by a photon beam must be identified. For this reason the treatment planning dose calculation is still only possible using CT. Newly developed algorithms, such as convolution/superposition and Monte Carlo, provide more accurate dose calculations by using heterogeneity correction [13]. These new algorithms calculate the radiation absorption and scatter of different tissue densities and apply that to the dose calculation, although this is only possible because the tissue density is obtained using a table that relates the Hounsfield number to density. Four-dimensional CT (4DCT) is another development used to quantify respiratory and organ motion. Normally, the 4DCT is applied in thoracic and abdominal sites in which respiratory motion can cause incorrect information regarding the size and position of a tumor and critical organs [14].

The primary disadvantage of CT for treatment planning is the low tissue contrast which can result in the tumor definition varying significantly from physician to physician [15]. Other imaging modalities, such as MR and PET, may help in the tumor definition due to their improved soft-tissue contrast and functional information [16, 17]. Therefore, in contemporary radiation therapy practice MR and PET are often used to complement CT for tumor delineation and normal tissue identification, although only CT is used for dose calculation. Therefore, the ability to accurately coregister these various image sets is one of the most powerful tools for radiation therapy planning.

### 3.1. MR

Paul Lauterbur, at the State University of New York, USA, and Peter Mansfield, at the University of Nottingham, England, independently produced the first NMR image, now called MR, in the 1970s. Their invention had a profound impact on medical imaging, and they shared the 2003 Nobel Prize in Physiology or Medicine.

MR is a powerful diagnostic tool. Compared to CT imaging, MR has several advantages, such as greater intrinsic soft-tissue contrast and resolution than CT and nonionizing radiation, as it uses radiofrequency waves for signal generation. In many clinical specialties, such as orthopedics, neurology, and neurosurgery, as well as for various anatomies and pathologies, including pelvic organs and tumors, soft-tissue visualization is used for diagnosis. Therefore, MR is the preferred imaging modality for many diagnostic imaging applications. Despite the wide use of MR for diagnostic imaging, in radiation therapy treatment planning MR is still a secondary image modality due to its image artifacts, lack of tissue density information, and relatively small field of view (FOV).

MR systems available only five years ago were less successful due to issues rendering them unsuitable for RT planning. However, improvements in MR hardware and software design have allowed MR imaging to become part of the RT planning workflow [18–21]. Protocols generated specifically for the purpose of RT planning rely on fundamentally robust, high-resolution, contrast-consistent, large FOV acquisitions, compared to the variety of sequences that may be used in diagnostic imaging. This effort has helped to increase the use of MR in RT planning, although still only as a secondary imaging set.

**Figure 5:**
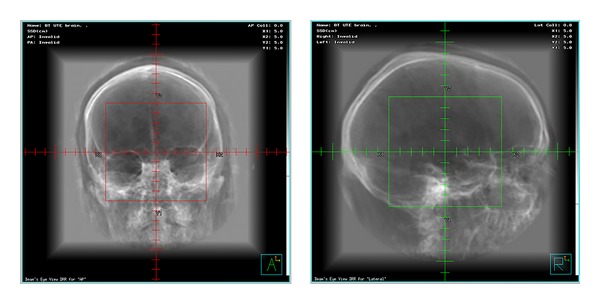
DRRs based solely on MR data.

Two, significant issues keep MR relegated to a secondary role, that is, the lack of electron-density information derived from MR images and the potential error in its geometric accuracy. Regarding electron density, therapy planning entails estimating the radiation dose which depends on the manner and degree to which the radiation interacts with and deposits energy in the tissue. This occurs primarily due to Compton events in which the incident, high-energy X-rays interact with outer shell electrons in the tissue. The interaction probability is proportional to electron density, on which the CT but not the MR signal depends. Although MR cannot directly image tissue density, there are several studies showing the potential to calculate the radiation dose and even generate DRRs based solely on MR data (Figure 5) [18, 20, 21]. A common approach is to assign a bulk density to the MR image using an atlas-based, electron-density mapping method [20, 22]. This remains an active area of research and new methods may be forthcoming.

Regarding the geometric accuracy, MR images may appear spatially warped so that the location of something appearing in the image differs from its actual physical location. This could be caused, for example, by distortions in the magnetic gradient fields. With MR, the spatial location is encoded by the spin frequency of protons which depends on the local magnetic-field strength. Magnetic gradients are used to vary the field strength in a spatially dependent manner. Spatial warping then results with aberrations in field strength that can be caused by residual error in system calibrations and by the presence of materials such as dental implants, prostheses and even due to transitions between materials, for example, between tissue and air.

To mitigate these effects one can use acquisition sequences that are less sensitive to magnetic field inaccuracies.  Table 2 shows the types of MR distortion, correction, and assessment. As mentioned above, current MR systems have hardware and software solutions available that optimize the geometric fidelity. Improved gradient linearity, static-field homogeneity, and patient-induced inhomogeneity compensation have brought MR greater acceptance for RT planning. Quality assurance tools to test various sources of image distortion can also be used to characterize these improvements on both a daily and a patient-by-patient basis. These advancements have increased the use of MR systems in radiation oncology clinical departments and have encouraged further development of MR-guided treatment solutions, including several projects intended to integrate MR imaging during external-beam radiation therapy [23–25].

### 3.2. PET

PET entails imaging the biodistribution of a radiolabeled compound selected based on its biochemical behavior. Most commonly, 2-[^18^F]fluoro-2-deoxy-D-glucose (FDG), a glucose analog, is used and is transported into cells, phosphorylated, and then trapped intracellularly. Differing from CT and conventional MR which show morphology, FDG-PET shows metabolically active tissue. As such, PET can be used to refine the target volume or to provide a dose boost to the most metabolically active tumors or areas therein [16]. PET can also be used to monitor tumor response, while noting that metabolic changes would precede changes in tumor shape and size [17].

While FDG is the most commonly used PET radiopharmaceutical, others may also be useful for radiation therapy. For example, some radiopharmaceuticals, such as ^64^Cu-diacetyl-bis(N^4^-methylthiosemicarbazone), commonly called ^64^Cu-ATSM, and ^18^F-fluoromisonidazole, commonly called ^18^F-FMISO, have been designed to demonstrate hypoxia, and as hypoxia is associated with radiation resistance, such areas may be targeted for additional radiation dosage [26]. 3′-[^18^F]fluoro-3′-deoxythymidine,^18^F-FLT has been developed as a marker of cellular proliferation and is used to assess the response to radiation therapy [27]. It has the potential to be an earlier indicator than FDG as radiation causes an inflammatory response that necessitates a delay between therapy and follow-up FDG-PET imaging [28].

**Figure 6:**
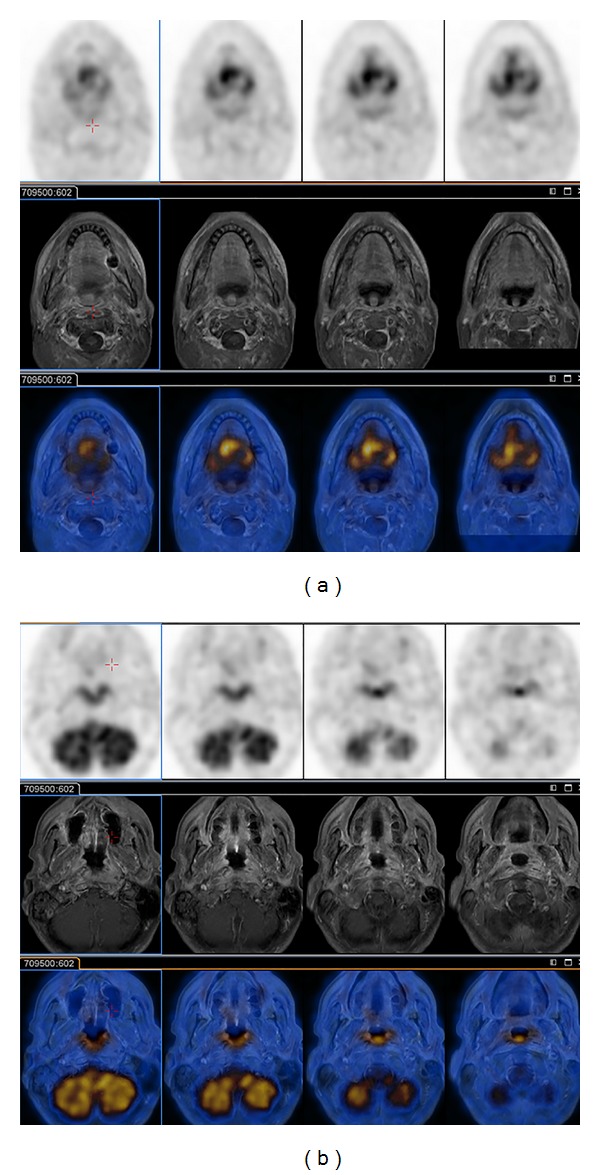
Axial-view images of a patient using a combined PET/MR scanner. The upper rows show PET images of FDG activity corresponding to glucose metabolism. The middle rows show MR images acquired using a T1-weighted, fat saturation sequence. The lower rows show the fused images. The PET images show elevated FDG uptake indicating cancer in (a), the tongue base and (b) the epiglottis.

Regardless of the radiotracer used, determining its spatial location is essential for radiation therapy planning. In this regard, image registration and fusion, that is, the ability to determine corresponding spatial locations in two or more image volumes and to visualize the result as a superpositioning of images, is a very useful technology. In the 1990s, image registration and fusion were achieved by scanning a patient on two, different scanners and then analyzing the data using a combination of computer software and human guidance to rotate and translate one of the two image volumes until it best matches the other. It is also possible to stretch or warp the image volumes to account for further differences in patient position and orientation, although it is less used and more prone to error than rigid-body transformations such as rotation and translation. Therefore, the advent of multimodality scanners, beginning around the year 2000, with combined PET-CT [29, 30], was a seminal advance for the use of imaging in radiation therapy planning as patients would be placed in the same position for both PET and CT. In fact, this change was so significant that the sale of PET scanners lacking CT absolutely disappeared within a few years. Motivated by the success of PET-CT, other combined instruments have been made, with PET-MR being the newest, and have the greatest number of technical challenges owing to the difficulty of operating PET detectors in the strong magnetic field of MR [31, 32]. Despite these and other challenges, it is exciting that MR offers soft-tissue contrast that is not possible with CT and that facilitates, for example, visualization of prostate and head and neck cancer (Figure 6).

### 3.3. Image Registration and Fusion

The ability to fuse and coregister the three, main types of oncologic imaging techniques, that is, CT, MR, and PET, became available for radiation oncology planning in the early 1990s due to the improvement of the software algorithms used to register and fuse multimodality imaging datasets. Registration is the ability to align the same points from different images. In medical applications, these points are the same anatomical regions of the body, such as bone and organs, for the same patient. Fusion is the ability to display different types of registered images anatomically overlain on one another in a single, composite image [33]. Fusion provides the best information for each image, that is, geometric definition and tissue density from the CT image, soft-tissue contrast from the MR image, and metabolic information from the PET image. The combined information reduces the uncertainty regarding the tumor definition for geometric localization as well as determining the size and spread of the disease. By improving the accuracy of the target definition, image fusion can potentially improve the treatment outcome and decrease complications as less normal tissue is irradiated.

Currently, most radiation treatment planning systems support image registration and fusion. There are several fusion algorithms. The most common is geometrical transformation (rigid and nonrigid) that can be point-based, intensity-based, or based on the mechanical properties of the tissue [34]. Registration algorithms are very complex and can create undesirable image artifacts, thus causing errors in the tumor and normal tissue localization. To minimize potential issues using software-based registration, it is desirable to position patients as similarly as possible and to use the software to refine the result. This can be achieved by using immobilization devices as well as using flat tables for diagnostic imaging equipment and which match the geometry of the treatment couch. As a patient's body may still move between and during the image set acquisitions, a 3-dimensional registration is necessary. Keeping the patient immobilized in the image set will produce smaller corrections for the fusion algorithm, thus causing fewer registration errors. Currently, the process of fusing multiple image techniques in radiation oncology is labor-intensive and requires manual verification of the quality of the registration by qualified experts [35]. Automation of this process will depend on improvements in the algorithms as well as better metrics for accuracy verification.

## 4. Future Considerations

The primary goal of treatment planning is to precisely calculate the radiation dose to the tumor in order to improve the outcome and reduce toxicity. The future of imaging in radiation therapy treatment planning is promising, and other advances will contribute to better target definition. Higher resolution imaging will be developed for all of the modalities discussed. Specifically, higher definition PET-CT scanners and high magnetic field MR have the ability to improve the visualization of tumors even to the level of microscopic disease extension [6].

Emerging algorithms for image fusion [33] will be more accurate and will allow an automated fusion process and verification. These new algorithms will make the tumor localization in the several types of images more precise and less time-consuming.

As previously mentioned, several solutions have been introduced in order to allow MR-based simulation and planning to become a reality. This may ultimately lead to the elimination of CT in radiation therapy, and which has been the foundation of treatment planning during the past four decades. This has far-reaching implications including lower overall cost and reduced X-ray exposure. However, another issue that has slowed the adoption of MR by radiation oncology is the lack of staff training for MR imaging and explaining the use of MR for treatment planning during traditional professional education.

Once MR systems are readily available for imaging patients undergoing radiation treatment, the opportunity to assess their response to treatment and to adapt the treatment plan for improved patient outcomes is likely [36–41]. The number and types of functional measurements which can be determined using MR imaging are multiple.  Table 3 shows some of the basic, functional imaging types that have shown promise in predicting the clinical outcomes for various tumor types.

### 4.1. “Omics” and the Future

Several investigators are attempting to apply the field of “omics” to tailor individual treatment for a better outcome in cancer therapy through the expression of genes, proteins, and metabolites. In radiation therapy “omics” may be able to predict the treatment response through immunohistochemical markers, DNA microarray gene signatures, and nucleotide polymorphisms [42]. Identifying biomarkers that can predict the sensitivity or resistance of tumors to radiation therapy is another promising area of ongoing research [43].

The advances in omics imaging for radiation treatment planning will include molecular imaging such as the new MR sequences described in Table 3, functional imaging, as well as the development and application of new PET tracers, such as ^18^F-FLT, ^64^Cu-ATSM, and ^18^F-FMISO, that can better identify regions of hypoxia, oxygen metabolism, microscopic disease, and high metabolism inside the tumor [27]. Tumor genetic and radiobiological factors will guide individualized radiation therapy with better target delineation, avoidance of normal tissue, dose escalation, dose fractionation, and better prediction of treatment response [44]. The semiquantitative, standardized uptake value (SUV) with different radiotracers for different tumor histologies will be able to predict the tumor heterogeneity based on metabolism. The SUV can identify more aggressive (metabolically active) or radioresistant (hypoxic) areas within a tumor and allow these areas to be treated with higher radiation doses (dose painting) [44].

In the omics era, therapy may be personally optimized based on pathologic and genetic characterization of tumors in order to target the relevant treatment pathways while minimizing undesirable side effects [45]. In both the past and present, detailed characterization requires biopsy. However, biopsy is invasive and only provides a snapshot of a subset of the cells of interest. As tumors are often heterogeneous, there may be multiple regions in a tumor, each with its own genetic profile. Tumors may be near nerves or other critical structures that make needle placement unacceptably risky. Molecular imaging has the promise to overcome these pitfalls while providing key insight regarding the tumor genetics, active pathways, and sensitivity to radiation, all as a spatial map which can be used to target a constellation of tumors and even regions within tumors [46].

In conclusion, the future of image-guided treatment planning is boundless and with continuous innovations that will ultimately lead to higher cure rates and less treatment-associated toxicity.

## Figures and Tables

**Table 1:** 

Accelerator voltage	Mean photon energy	Classification
10–150 kV	3–50 keV	Superficial
150–500 kV	50–166 keV	Orthovoltage
500–1000 kV	166–333 keV	Supervoltage
>1000 kV (1 MV)	>333 keV (0.33 MeV)	Megavoltage

**Table 2:** Sources of MR distortion, correction, and method for assessment.

Level of distortion	Distortion source	Distortion correction	Assessment of residual distortion
System	Gradient nonlinearity	Gradient coil design (HW) Model-based corrections (SW)	Geometric fidelity phantoms and assessment tools
	Static field (B0) inhomogeneity	Magnet design and static-field shims (HW)	B0-field map in phantoms

Patient	Static-field (B0) inhomogeneity	Gradient-shim coils (HW) Patient-specific B0 field correction (SW)	B0-field map in the patient
	Transmit-field (B1) inhomogeneity	Multiple RF transmit channels (HW) Patient-specific B1 field correction (SW)	B1-field map in the patient

Sequence	Gradient switching (eddy currents)	Gradient preemphasis (HW/SW) Protocols with low gradient slew rates (SW)	Visual inspection

**Table 3:** Basic MR functional imaging types that have shown promise in predicting outcomes for various tumor types.

Type of measurement	Functional imaging method	Known as	What is measured
Perfusion	Dynamic contrast enhanced	DCE, permeability	Gadolinium-induced shortening of T1
	Dynamic susceptibility contrast	DSC	Gadolinium-induced shortening of T2*
	Arterial spin labeling	ASL	Intrinsic contrast enhancement generated from magnetization of arterial blood

Diffusion	Diffusion weighting imaging	DWI	Gradient-induced sensitization of molecular diffusion

Metabolic function	Spectroscopy	MRSI	Chemical composition based on resonant frequency

Oxygenation	Bold-level oxygen dependent	BOLD, fMRI	T2* differences in oxy- and deoxyhemoglobin
